# New Hybrids Based on Curcumin and Resveratrol: Synthesis, Cytotoxicity and Antiproliferative Activity against Colorectal Cancer Cells

**DOI:** 10.3390/molecules26092661

**Published:** 2021-05-01

**Authors:** Cristian Hernández, Gustavo Moreno, Angie Herrera-R, Wilson Cardona-G

**Affiliations:** Química de Plantas Colombianas, Faculty of Exact and Natural Sciences, Institute of Chemistry, University of Antioquia (UdeA), Calle 70 No. 52–21, Medellín AA 1226, Colombia; cristian.hernandezs@udea.edu.co (C.H.); gustavo.morenoq@gmail.com (G.M.); angie.herrerar@udea.edu.co (A.H.-R.)

**Keywords:** curcumin, resveratrol, hybrid molecules, colorectal cancer, cytotoxicity, antiproliferative activity

## Abstract

We synthesized twelve hybrids based on curcumin and resveratrol, and their structures were elucidated by spectroscopic analysis. The chemopreventive potential of these compounds was evaluated against SW480 human colon adenocarcinoma cells, its metastatic derivative SW620, along with the non-malignant CHO-K1 cell line. Among the tested compounds, hybrids **3e** and **3i** (for SW480) and **3a**, **3e** and **3k** (for SW620) displayed the best cytotoxic activity with IC_50_ values ranging from 11.52 ± 2.78 to 29.33 ± 4.73 µM for both cell lines, with selectivity indices (SI) higher than 1, after 48 h of treatment. Selectivity indices were even higher than those reported for the reference drug, 5-fluorouracil (SI = 0.96), the starting compound resveratrol (SI = 0.45) and the equimolar mixture of curcumin plus resveratrol (SI = 0.77). The previous hybrids showed good antiproliferative activity.

## 1. Introduction

Colorectal cancer (CRC) is the third most diagnosed malignancy and the fourth leading cause of cancer-related death worldwide. Despite the progress in diagnosis and treatment, incidence and mortality of CRC cancer is unacceptably high [1]. Current chemotherapy is based on 5-fluorouracil, oxaliplatin and irinotecan, which, although effective, induce severe side effects to the patients; thus, novel approaches for the treatment of colorectal cancer are needed [2].

Curcumin is one of the bioactive components of *Curcuma longa* Linn. Several studies with this compound have revealed important functions in cancer control [3], including antiproliferative activity against different cancer cell lines and inhibition at different stages of cancer cell progression [4]. Various growth factor receptors and cell adhesion molecules involved in tumor growth, metastasis, apoptosis and multidrug resistance are affected by curcumin [5,6,7]. Resveratrol has also received great interest in drug discovery mainly due to its various biological activities including antitumor [8,9], affecting all three stages of carcinogenesis (initiation, promotion and progression) by modulating signal transduction pathways that control cell division and growth, apoptosis, inflammation, angiogenesis and metastasis [10,11,12,13,14,15,16]. Different studies have shown the importance of hydroxyl group for the biological activity; however, resveratrol analogs containing methoxy groups have increased the activity [8,9,17]. Both curcumin and resveratrol alone have shown restricted clinical efficacy due to different factors, including low bioavailability and stability stands out [18,19].

The synthesis of curcumin and resveratrol hybrids has emerged as a promising strategy in recent drug discovery research to develop highly active anticancer therapies. The hybrid anticancer drug approach is an innovative synthetic strategy that combines two or more bioactive scaffolds with relevant pharmacological action [20,21], where the presence of different pharmacophores in a single unit could improve the ability to inhibit more than one biological target, displaying a synergistic effect in biological activity. The goal of the molecular hybridization approach is to improve anticancer activity and selectivity with a subsequent reduction in side effects regarding conventional chemotherapy [18].

**Figure 1:**
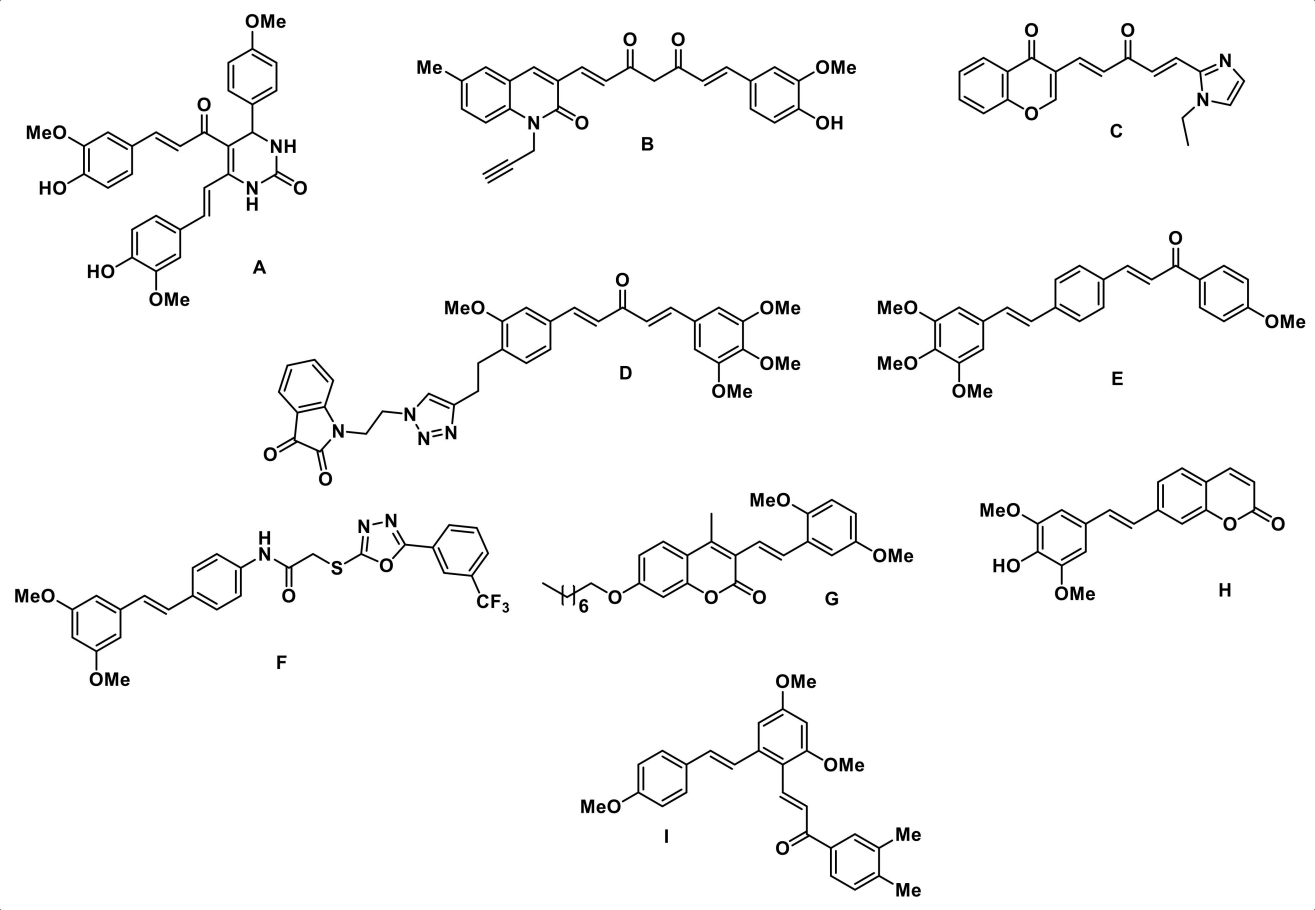
Hybrid molecules derived from curcumin and resveratrol with anticancer activity.

Curcumin–monastrol hybrid (Figure 1A) showed maximum activity with a mean growth percentage (GP) of 88.90; besides, it was found to be the most sensitive on MDA-MB-231/ATCC (breast cancer), PC-3 (prostate cancer), SNB-75 (CNS cancer), RPMI-8226 (leukemia), MOLT-4 (leukemia), CCRF-CEM (leukemia) and HS 578T (breast cancer) cell lines with GP values of 58.50, 59.60, 60.07, 64.11, 72.84 and 73.39, respectively [22]. On the other hand, curcumin–quinolone hybrid (Figure 1B) was evaluated against two human lung cancer cells (A549 and H-460), with IC_50_ values of 23.9 and 21.75 µM, respectively; the MCF-7 human breast cancer cell line (IC_50_ values of 36.2 µM); and SKOV-3 ovarian carcinoma cells (IC_50_ value of 12.8 µM) with selectivity against SKOV-3 cell line, inducing changes in normal cell morphology and apoptosis [23]. Hybrid curcum–imidazol (Figure 1C) showed antiproliferative activity against LNCaP (human androgen-dependent prostate cancer) cell line with an IC_50_ value of 1.0 µM [24]. 1H-1,2,3-triazole tethered curcumin–isatin hybrid (Figure 1D) was tested against a panel of cancer cell lines exhibiting potent activity with IC_50_ values ranging from 1.12 to 5.67 µM. Moreover, this compound showed inhibition of tubulin polymerization at 1.2 µM when evaluated in HCT-116 cells [25]. Hybrid stilbene–chalcone (Figure 1E) showed high selectivity towards certain ovarian cancer, non-small cell lung cancer and breast cancer cell lines with GI_50_ values in the range of 1.28–34.1 µM [26]. Resveratrol–oxadiazole hybrid F showed great activity against SiHa, MDA-MB-231 and PANC-1 human cancer cells (GI_50_ < 0.1 µM), being superior to the reference compound resveratrol [27]. Hybrids based on stilbene–coumarin (Figure 1G,H) exhibited selective activity against SW480 cells (IC_50/_48 h = 6.92 and SI/48 h ≥ 400 for compound Figure 1G; IC_50/_48 h = 1.01 µM and SI/48 h = 67.8 for compound Figure 1H). In addition, these compounds were able to preserve their activities over time. The results achieved by these hybrids are even better than the lead compounds (coumarin and resveratrol) and the standard drug (5-FU) [28]. In addition, compound Figure 1I, a resveratrol–chalcone hybrid, exhibited potent in vitro activity, causing growth inhibition of HepG2, B16-F10 and A549 cell lines, with IC_50_ values of 0.2, 0.1 and 1.4 µg/mL, respectively. This hybrid also exhibited significant tubulin polymerization inhibitory activity at 2.6 µg/mL [29] (Figure 1).

**Figure 2:**
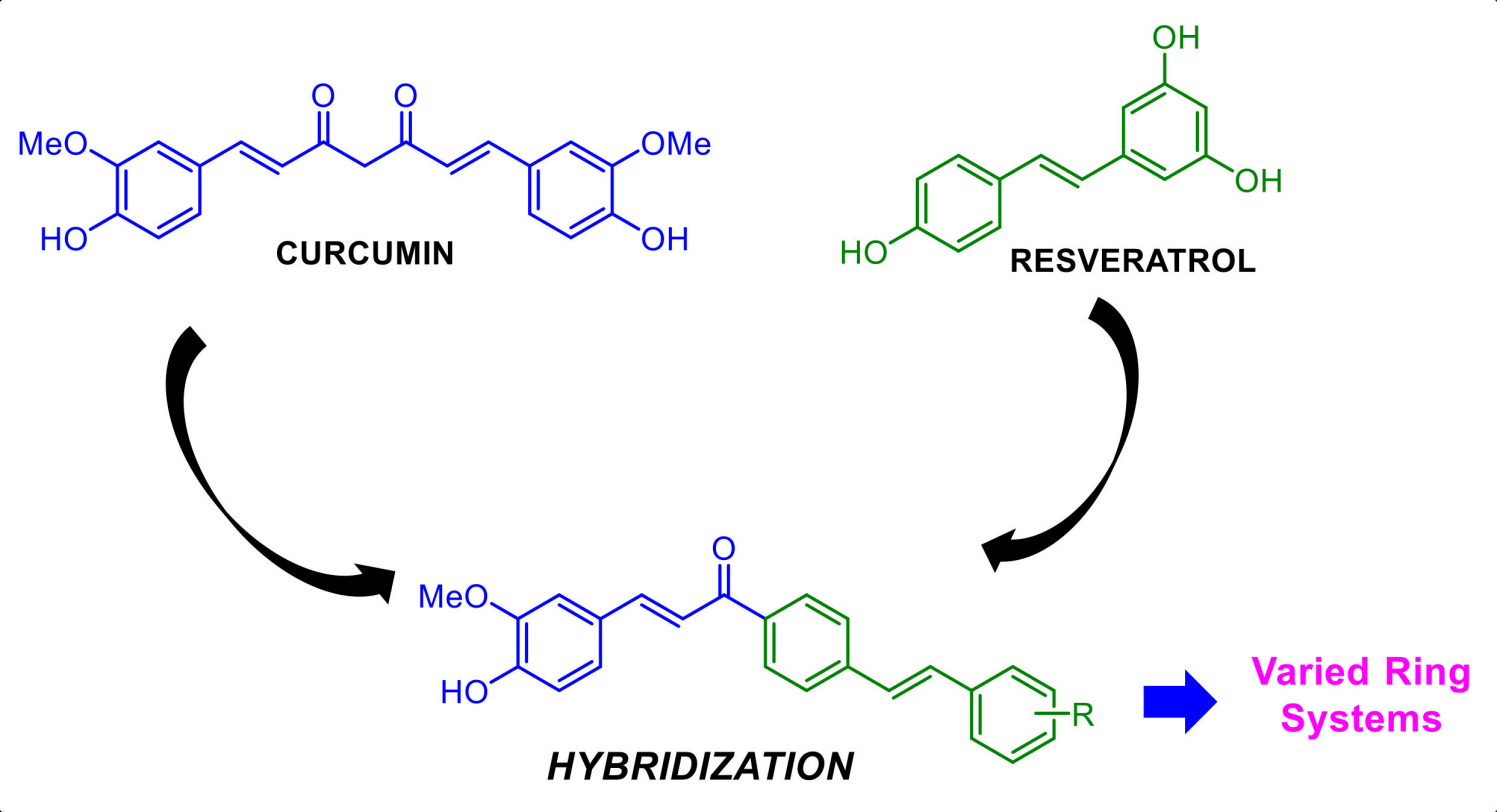
Design of hybrids based on curcumin and resveratrol as anti-cancer agents.

We synthesized several hybrids based on curcumin and resveratrol (Figure 2), and the cytotoxicity and antiproliferative effect were determined to identify possible therapeutic approaches for the treatment of colorectal cancer.

## 2. Results and Discussion

### 2.1. Chemistry

**Scheme 1:**
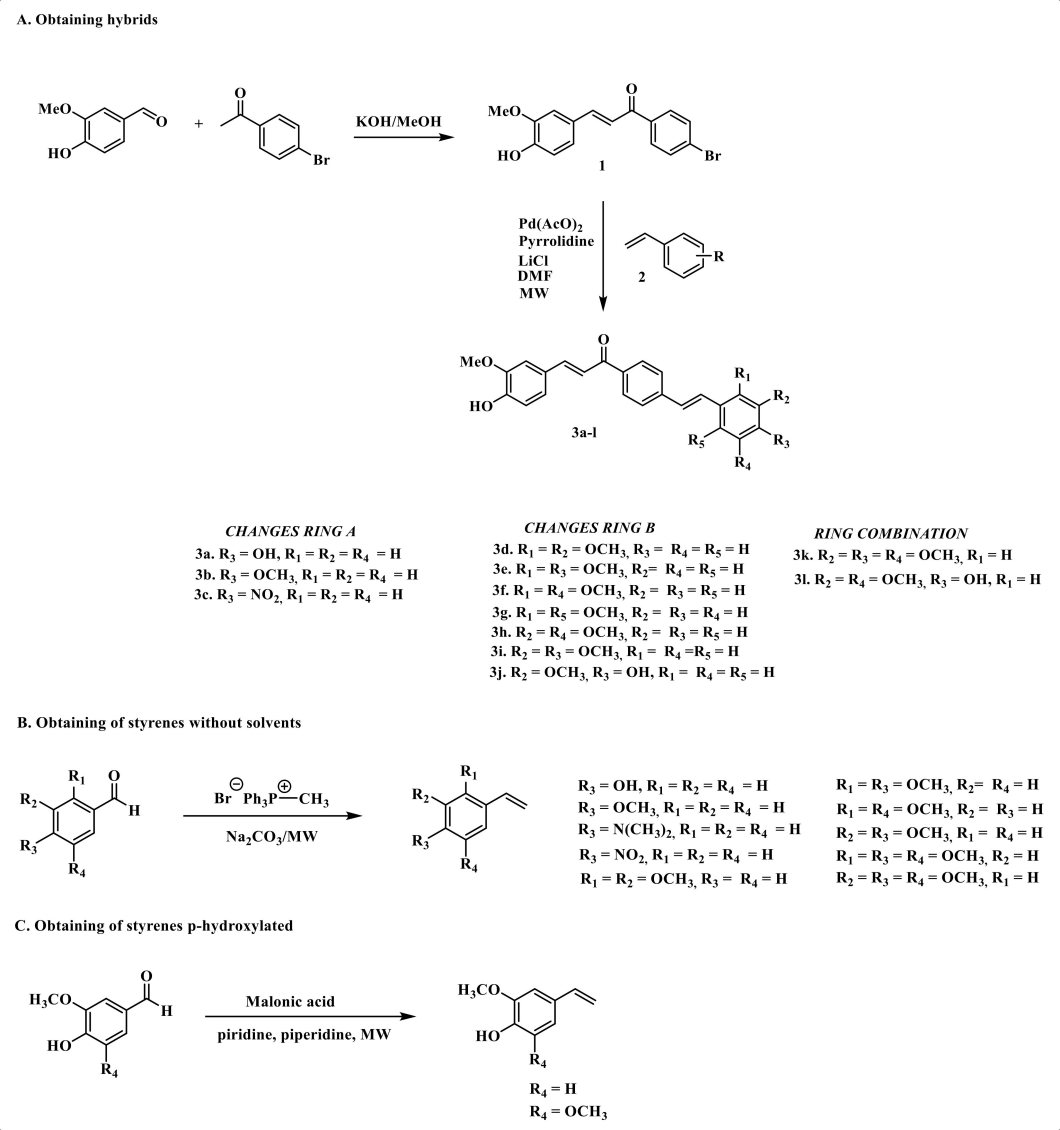
Synthesis of hybrids based on curcumin and resveratrol.

In this study, the synthesis of the hybrids began with obtained chalcone **1** by mean the Claisen–Schmidt condensation and ultrasonic irradiation assisted between 4-bromoacetotophenone and vanillin (yield 63%) [30]. This compound has already been reported [31], and it was subjected to cross-coupling reaction with various styrenes (**2**) under palladium catalysis [32], leading to the formation of hybrids **3a**–**3l** with 35–55% yields. To improve these results, other methodologies were tested such as (Pd(Ph_3_)_4_, AgOAc, DMF) [33], Pd(AcO)_2_, PPh_3_, CH_3_CO_2_Na [34], Pd(AcO)_2_ and triethanolamine [28], but these results were not as expected. Styrenes (**2**) were synthesized via microwave assisted Wittig reaction between substituted aldehydes and methyltriphenylphosphonium bromide [28,35] and Knoevenagel condensation reactions between 4-hydroxybenzaldehydes with malonic acid [28,36] (Scheme 1).

The structures of all compounds were established by a combined study of IR, ESI-MS, ^1^H-NMR, ^13^C-NMR and COSY spectra. IR spectra exhibited characteristic absorption peaks corresponding to C = O, C = C, C = CAr and C-HAr. ESI-MS spectra showed characteristic [M+H]^+^ peaks corresponding to the molecular weights. The signals of H or C atoms were performed using typical δ-values and coupling constants (*J*). The ^1^H-NMR spectra of hybrids dissolved in CDCl_3_ showed signals of Ar-CH (~8.00 ppm, *d*, 2H), Ar-CH (~7.60 ppm, *d*, 2H), H-C = C-H_trans_ (~7.77 and 7.40 ppm) and -OCH_3_ (3.80–3.90 ppm). ^13^C-NMR spectra of hybrids showed signals around 56.28, 128, 129, 145 and 189 ppm, corresponding to -OCH_3_, -C = C-, Ar-CH (x2), -C = C- and C = O, respectively.

### 2.2. Biological Activity

#### 2.2.1. Cytotoxic Effect of Hybrids Based on Curcumin and Resveratrol on SW480, SW620 and CHO-K1 Cell Lines

To determine the cytotoxic effect of the synthesized hybrids, they were evaluated against SW480 human colon adenocarcinoma cells, its metastatic derivative SW620 and the non-malignant CHO-K1 cell line, through the sulforhodamine B assay. In this experiment, 5-FU was also included as the reference drug, as well as the parental subunits (curcumin and resveratrol) and the equimolar mixture of these. Cytotoxicity was reported as 50% inhibitory concentration (IC_50_ values). All results regarding the cytotoxic effect are summarized in Table 1.

Among the tested compounds, it was observed that hybrids **3e** and **3i** (for SW480), together with **3a**, **3e** and **3k** (for SW620), displayed the best cytotoxic activity with IC_50_ values ranging from 11.52 ± 2.78 to 29.18 ± 4.36 µM, after 48 h of treatment. These compounds demonstrate the objective of molecular hybridization, improving the biological activity, since they were more active than parental compounds. They were also more selective than the equimolar mixture and the reference drug. These results are in accordance with those previously reported by Castrillon and colleagues (2019) [37], who also reported cytotoxic and selective activity of different *S*-allyl cysteine hybrids, using the same model. Moreover, similar results were reported by Herrera-R et al. (2018) [28], who synthesized another class of hybrid compounds using different scaffolds as coumarin and stilbene (styrylcoumarin), finding better cytotoxic activity and selectivity when tested using an in vitro model of colorectal cancer (CRC). Furthermore, compounds **3c**, **3g** and **3j** decreased activity after 48 h of treatment, as evidenced by the increase of the IC_50_ value compared to 24 h, for both cell lines. On the other hand, although hybrids **3d**, **3h** and **3l** improved the activity over malignant cells (SW480 and SW620) after 48 h of treatment, a decrease in selectivity was also observed due to the high toxicity on non-malignant cells. Compound **3b** was not successfully evaluated due to solubility problems.

In the structure–activity relationship (SAR) study at 48 h, we noticed a synergistic action of the parent subunits when they are linked to form a single structure in the hybrid. The same result was displayed over SW480 cell line by other hybrids [28,37]. When we compared compounds **3a** and **3b** with **3c**, it was observed that electron donor groups favored activity unlike electron withdrawing groups. Except for compounds **3d** and **3i**, all di-oxygenated compounds (**3d**–**3j**) showed better activity over SW620 cell line than SW480 cells. These hybrids exhibited similar activity (20–29 µM) on SW620 cell line. Hybrid **3i**, which has oxygenated positions 3 and 4, showed the best activity on SW480. The **3k** hybrid, a combination of compounds **3b** and **3h**, exhibited a synergistic effect on SW620 cells reflected in increased activity when compared to initial hybrids. In both mono-oxygenated and di-oxygenated compounds, it was observed that those with hydroxyl groups exhibited less activity than methoxylated compounds. The importance of hydroxyl groups in compounds such as chalcones, coumarins, caffeic acid esters and allylcysteine–caffeic acid hybrids [38,39,40,41] is related with better molecular recognition ability towards target bioreceptors upon hydrogen bond formation [42], oxidation processes across radical formation and/or the ability of metal complexation [43]. However, considering that our compounds with hydroxyl groups were less active, this suggests they could probably have different mechanism of action. The results of these compounds are in agreement with other reports for several methoxy derivatives of resveratrol which were evaluated against SW480 or KB lines [44,45,46].

#### 2.2.2. Antiproliferative Effect of Hybrids Based on Curcumin and Resveratrol on SW480 and SW620 Cells

**Figure 3:**
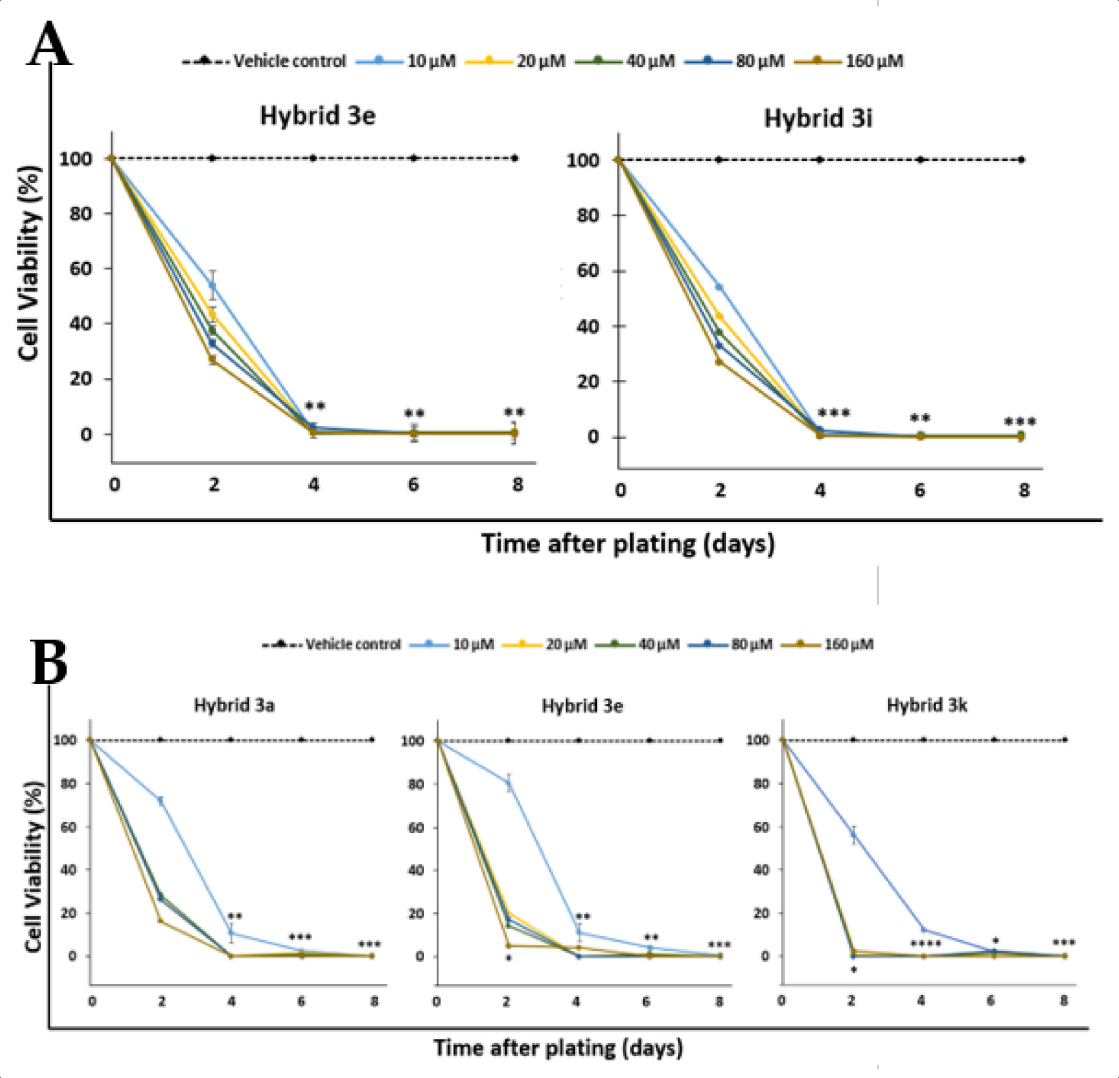
Antiproliferative effect of hybrids based on curcumin and resveratrol against: SW480 cell line (**A**); and its metastatic derivative SW620 cell line (**B**). Data are presented as the mean ± SE of at least three independent experiments (* *p* < 0.05; ** *p* < 0.01; *** *p* < 0.001; **** *p* < 0.0001). Vehicle control was assumed as 100% of viability.

With the aim of testing the antiproliferative effect of the most cytotoxic and selective hybrids (**3e** and **3i** for SW480 and **3a**, **3e** and **3k** for SW620), they were analyzed through a longer period. According to the results in comparison with the control, it was observed that the activity was time- and concentration-dependent, as evidenced by the decrease in cell viability in the highest concentrations (Table 2). Among the results, it was observed that all compounds evaluated displayed significant antiproliferative activity and, thus, viability percentages were reduced (*p* values < 0.01) in both SW480 and SW620 cells after four days of treatment. Besides, in SW620 cells, hybrids **3e** and **3k** displayed significant reduction in viability, after just two days of treatment with concentrations higher than 10 µM (Figure 3A,B). In addition, when treated cells were observed with optical microscope, the cellular morphology was severely perturbed, exhibiting changes in size and shape after treatment. Similar results were presented in a previous investigation by Herrera-R et al. (2018) [28], reporting the antiproliferative effect and morphological changes in SW480 cells after treatment with different hybrids using a stilbene moiety, which highlights the potential of this scaffold in the approach to design molecules with potential activity against CRC.

## 3. Conclusions

We showed for the first time that the synthesized hybrids based on curcumin and resveratrol have chemopreventive potential in an in vitro model of CRC (using two different adenocarcinoma cells), involving selective growth inhibition and antiproliferative activity, with changes in size and shape after treatment, better results than parental compounds and exhibiting higher selectivity than the conventional chemotherapeutic (5-FU) and the equimolar mixture, highlighting the potential of the strategy based on molecular hybridization. Our findings suggest that these hybrid molecules could be promising chemopreventive agents against colorectal cancer, thus it is necessary to carry out further studies.

## 4. Experimental Section

### 4.1. Chemical Synthesis

#### 4.1.1. General Remarks

Microwave reactions were carried out in a CEM Discover microwave reactor in sealed vessels (monowave, maximum power 300 W, temperature control by IR sensor and fixed temperature). IR spectra were recorded on a Spectrum RX I FT-IR system (Perkin-Elmer, Waltham, MA, USA) in KBr disks. ^1^H- and ^13^C-NMR spectra were recorded on a Varian instrument operating at 300 and 75 MHz, respectively. The signals of the deuterated solvent (CDCl_3_ or DMSO-D_6_) were used as reference. Chemical shifts (δ) are expressed in ppm with the solvent peak as reference and TMS as an internal standard; coupling constants (J) are given in Hertz (Hz). Carbon atom types (C, CH, CH_2_ and CH_3_) were determined using the DEPT pulse sequence. HRMS was obtained using a Bruker Impact II UHR-Q-TOF mass spectrometry (Bruker Daltonik GmbH, Bremen Germany) in positive mode. Silica gel 60 (0.063–0.200 mesh, Merck, Whitehouse Station, NJ, USA) was used for column chromatography and precoated silica gel plates (Merck 60 F254 0.2 mm) were used for thin layer chromatography (TLC). Monitoring of the reaction progress and product purification was carried out by TLC.

#### 4.1.2. General Procedure for the Synthesis of (E)-1-(4-Bromophenyl)-3-(4-Hydroxy-3-Methoxyphenyl)prop-2-en-1-one (1)

This compound was prepared by the condensation of 4-bromoacetophenone 1 (10 mmoL, 2.0 g) and vanillin 2 (10 mmoL, 1.52 g) in a solution of 20% KOH in ethanol (20 mL). The reaction mixture was sonicated for 1 h and neutralized with a solution of 10% HCl. The solid was filtered, sequentially washed with water, dried and recrystallized from ethanol to obtain the corresponding chalcone in 63% yield (6.3 mmoL, 2.1 g).

#### 4.1.3. Styrene Synthesis

*Method 1:* Wittig Reaction under microwave conditions.

Potassium carbonate (0.10 mol) and (ethyl)triphenylphosphonium iodide (0.016 mol) were mixed and crushed in a mortar to a fine powder and transferred into a 250 mL Erlenmeyer flask. Aldehyde (0.016 mol) was added to the powdered mixture and thoroughly mixed. The Erlenmeyer flask was covered with a watch glass and placed into a microwave along with a 1 L beaker of ice (to ensure that the reaction mixture did not overheat). The microwave was run at full power for 12 min, making sure to stop the heating and replace the ice when it had melted. A tiny sample of the crude reaction product was removed, diluted in a small amount of hexanes and analyzed by TLC to check the reaction progress. Upon completion, the crude solids were extracted with hexanes (3 × 20 mL). The combined hexane extracts were placed in a round-bottomed flask and concentrated by rotary evaporation to obtain a yellow oil which was purified by flash chromatography on silica gel using hexanes or a mixture of hexanes/ethyl acetate as eluent.

*Method 2:* Knoevenagel condensation reactions:

Piperidine (0.75 mL, 7.6 mmoL) was added to a solution of aldehyde (5 mmoL) and malonic acid (20 mmoL) in pyridine (21 mL). The mixture was heated at reflux for a period of 30 min under microwave irradiation. Then, toluene (40 mL) was added to the cooled reaction mixture and the solvent volume reduced in vacuum at 30–40 °C. Additional toluene (20 mL) was then added and the solvent again removed in vacuum to eliminate all traces of pyridine. This procedure afforded a residue which was purified by column chromatography over silica gel. Elution with 9:1 hexane/ethyl acetate afforded the corresponding vinylphenol.

#### 4.1.4. General Procedure for the Mizoroki-Heck Reactions

Styrene (0.3 mmoL), bromochalcone (0.3 mmoL, 100 mg), pyrrolidine (0.6 mmoL, 50 µL), Pd(OAc)_2_ (6 mg, 0.027 mmoL), LiCl (12 mg, 0.28 mmoL) and DMF (1 mL) were placed in a 10 mL flat-bottomed flask equipped with a magnetic stirring bar. The mixture was stirred and heated to 170 °C at 200 W for a period of 4 min, under microwave irradiation. The mixture of reaction was allowed to cool, and then 100 mL of HCl 5% were added. Then, this mixture was extracted with dichloromethane, concentrated and the crude residue was purified by column chromatography (silica gel, hexane-ethyl acetate mixture) affording the coupling products in 35–55% yield.

(E)-3-(4-Hydroxy-3-methoxyphenyl)-1-(4-((E)-4-hydroxystyryl)phenyl)prop-2-en-1-one (**3a**)

Yield 20%; m.p. 119–120 °C; IR (cm^−1^): ν_max_ 3271 (OH), 1645 (C = O), 1593 (C = C), 1512 (C = C_Ar_), 1267 (C-O-C). ^1^H-NMR (300 MHz, DMSO-*d6*) δ 8.13 (d, *J* = 8.1 Hz, 2H), 7.80–7.62 (m, 4H), 7.53–7.45 (m, 3H), 7.35 (d, *J* = 16.4 Hz, 1H), 7.27 (dd, *J* = 8.1, 2.0 Hz, 1H), 7.13 (d, *J* = 16.4 Hz, 1H), 6.80 (dd, *J* = 8.1, 2.0 Hz, 3H), 3.87 (s, 3H). ^13^C-NMR (75 MHz, DMSO-*d6*) δ 188.32 (C = O), 158.51 (Ar-O), 151.50 (Ar-O),148.72 (Ar-O), 145.26, 142.52 (C = C), 136.69 (Ar), 131.72 (Ar), 129.44 (2C Ar), 128.87 (2C Ar), 128.09 (Ar), 126.57 (2C Ar), 126.01 (Ar), 124.97 (Ar), 124.51 (C = C), 118.40 (Ar), 116.23 (Ar), 116.16 (2C Ar), 112.03 (Ar), 56.23 (OCH_3_). HRMS-ESI (*m*/*z*): 373,1435 [M+H]^+^ calcd. for C_24_H_21_O_4_ [M + H]^+^ 373,1361

(E)-3-(4-hydroxy-3-methoxyphenyl)-1-(4-((E)-4-methoxystyryl)phenyl)prop-2-en-1-one (**3b**)

Yield 50%; m.p. 187–190 °C; IR (cm^−1^): ν_max_ 3248 (OH), 1641 (C = O), 1568 (C = C), 1452 (C = C_Ar_), 1263 (C-O-C); ^1^H-NMR (300 MHz, DMSO-*d6*) δ 8.15 (d, *J* = 8.2 Hz, 2H), 7.78 (d, *J* = 15.4 Hz, 1H), 7.74 (d, *J* = 8.2 Hz, 2H), 7.69 (d, *J* = 15.4 Hz, 1H), 7.61 (d, *J* = 8.6 Hz, 2H), 7.52 (d, *J* = 1.4 Hz, 1H), 7.41 (d, *J* = 16.5 Hz, 1H), 7.30 (dd, *J* = 8.2, 1.4 Hz, 1H), 7.22 (d, *J* = 16.5 Hz, 1H), 6.98 (d, *J* = 8.6 Hz, 2H), 6.84 (d, *J* = 8.2 Hz, 1H), 3.88 (s, 3H), 3.80 (s, 3H). ^13^C-NMR (75 MHz, DMSO) δ 188.42 (C = O), 159.90 (Ar-O), 150.45 (Ar-O), 148.50 (Ar-O), 145.16 (C = C), 142.35 (Ar), 136.78 (Ar), 131.31 (C = C), 129.76 (C = C), 129.47 (2C Ar), 129.05 (Ar), 128.75 (2C Ar), 126.73 (Ar), 126.62 (Ar), 125.58 (Ar), 124.70 (C = C), 118.90 (Ar), 116.07 (Ar), 114.73 (2C Ar), 114.36 (Ar), 112.12 (Ar), 56.28 (OCH_3_), 55.66 (OCH_3_). HRMS-ESI (*m*/*z*): 387,1589 [M+H]^+^ calcd. for C_25_H_23_O_4_ [M + H]^+^ 387.1518

(E)-3-(4-hydroxy-3-methoxyphenyl)-1-(4-((E)-4-nitrostyryl)phenyl)prop-2-en-1-one (*3c*)

Yield 35%; m.p. 227–230 °C; IR (cm^−1^): ν_max_ 3232 (OH), 1643 (C = O), 1556 (C = C), 1452 (C = C_Ar_), 1303 (NO_2_), 1276 (C-O-C); ^1^H-NMR (300 MHz, DMSO-*d6*) δ 8.13 (d, *J* = 8.4 Hz, 2H), 7.81 (d, *J* = 15.5 Hz, 1H), 7.73–7.63 (m, 3H), 7.53 (d, *J* = 1.5 Hz, 1H), 7.49 (d, *J* = 8.8 Hz, 2H), 7.34 (d, *J* = 16.6 Hz, 1H), 7.30 (dd, *J* = 8.1, 1.5 Hz, 1H), 7.08 (d, *J* = 16.6 Hz, 1H), 6.85 (d, *J* = 8.1 Hz, 1H), 6.73 (d, *J* = 8.8 Hz, 2H), 3.88 (s, 3H). ^13^C-NMR (75 MHz, DMSO) δ 188.30 (C = O), 150.81 (Ar-O), 150.13 (Ar-O), 148.45 (Ar-NO_2_), 144.93 (C = C), 145.02 (Ar), 142.99 (Ar), 136.17 (Ar), 132.13 (Ar), 129.50 (C = C), 128.55 (2C Ar), 126.83 (C = C), 126.42 (Ar), 126.31 (Ar), 124.84 (Ar), 124.58 (C = C), 122.81 (Ar), 119.07 (Ar), 116.04 (Ar), 112.58 (2C Ar), 112.11 (Ar), 56.29 (OCH_3_). HRMS-ESI (*m*/*z*): 402,1977 [M+H]^+^ calcd. for C_24_H_20_NO_5_ [M + H]^+^ 402.1263

(E)-1-(4-((E)-2,3-Dimethoxystyryl)phenyl)-3-(4-hydroxy-3-methoxyphenyl)prop-2-en-1-one (**3d**)

Yield 55%; m.p. 63–65 °C; IR (cm^−1^): ν_max_ 3359 (OH), 1651 (C = O), 1575 (C = C), 1510 (C = C_Ar_), 1267 (C-O-C); ^1^H-NMR (600 MHz, CDCl_3_) δ 8.03 (d, *J* = 8.1 Hz, 2H), 7.77 (d, *J* = 15.5 Hz, 1H), 7.66 (d, *J* = 8.1 Hz, 2H), 7.59 (d, *J* = 16.4 Hz, 1H), 7.40 (d, *J* = 15.5 Hz, 1H), 7.27 (d, *J* = 8.3 Hz, 1H), 7.23 (d, *J* = 8.3 Hz, 1H), 7.18 (d, *J* = 16.4 Hz, 1H), 7.14 (s, 1H), 7.08 (t, *J* = 8.0 Hz), 6.96 (d, *J* = 8.3 Hz, 1H), 6.88 (d, *J* = 8.0 Hz, 1H), 3.97 (OCH_3_), 3.90 (OCH_3_), 3.88 (OCH_3_). ^13^C-NMR (75 MHz, Chloroform-d) δ 189.79 (C = O), 153.17 (Ar-O), 148.36 (Ar-O), 147.27 (Ar-O), 146.87 (Ar-O), 145.07 (C = C), 142.00 (Ar), 137.32 (Ar), 131.03 (Ar), 129.06 (2C Ar), 128.84 (C = C), 127.59 (Ar), 126.75 (2C Ar), 125.61 (C = C), 124.28 (C = C), 123.45 (Ar), 119.71 (Ar), 117.99 (Ar), 114.95 (Ar), 111.99 (Ar), 110.07 (Ar), 61.29 (OCH_3_), 56.07 (OCH_3_), 55.89 (OCH_3_). HRMS-ESI (*m*/*z*): 417,1696 [M+H]^+^ calcd. for C_26_H_25_O_5_ [M + H]^+^ 417.1623

(E)-1-(4-((E)-2,4-Dimethoxystyryl)phenyl)-3-(4-hydroxy-3-methoxyphenyl)prop-2-en-1-one (**3e**)

Yield 40%; m.p. 78–82 °C; IR (cm^−1^): ν_max_ 3349 (OH), 1649 (C = O), 1575 (C = C), 1508 (C = C_Ar_), 1259 (C-O-C); ^1^H-NMR (600 MHz, CDCl_3_) δ 7.99 (d, *J* = 7.9 Hz, 2H), 7.76 (d, *J* = 15.5 Hz, 1H), 7.60 (d, *J* = 7.9 Hz, 2H), 7,53 (d,1H, *J* = 15.5 Hz), 7,53 (d, 1H, *J* = 8.5 Hz), 7.40 (d, *J* = 15.5 Hz, 1H), 7.22 (d, *J* = 7.8 Hz, 1H), 7.13 (s, 1H), 7.06 (d, *J* = 15.5 Hz, 1H), 6.95 (d, *J* = 7.9 Hz, 1H), 6.53 (d, *J* = 8.5 Hz, 1H), 6.48 (s, 1H), 4.60 (OH), 3.95 (OCH_3_), 3.88 (OCH_3_), 3.84 (OCH_3_). ^13^C-NMR (75 MHz, Chloroform-d) δ 189.77 (C = O), 161.13 (Ar-O), 158.42 (Ar-O), 148.31 (Ar-O), 146.88 (Ar-O), 144.85 (C = C), 142.85 (Ar), 136.63 (Ar), 129.04 (2C Ar), 127.69 (C = C), 127.64 (Ar), 126.31 (2C Ar), 126.09 (C = C), 125.80 (C = C), 123.37 (Ar), 119.74 (Ar), 118.98 (Ar), 114.96 (Ar), 110.12 (Ar), 105.17 (Ar), 98.51 (Ar), 56.06 (OCH_3_), 55.59 (OCH_3_), 55.50 (OCH_3_). HRMS-ESI (*m*/*z*): 417,1696 [M+H]^+^ calcd. for C_26_H_25_O_5_ [M + H]^+^ 417.1623

(E)-1-(4-((E)-2,5-Dimethoxystyryl)phenyl)-3-(4-hydroxy-3-methoxyphenyl)prop-2-en-1-one (**3f**)

Yield 38%; m.p. 47–51 °C; IR (cm^−1^): ν_max_ 3379 (OH), 1651 (C = O), 1577 (C = C), 1508 (C = C_Ar_), 1265 (C-O-C); ^1^H-NMR (300 MHz, CDCl_3_) δ 8.01 (d, *J* = 8.3 Hz, 2H), 7.77 (d, *J* = 15.6 Hz, 1H), 7.63 (d, *J* = 8.3 Hz, 2H), 7.56 (d, *J* = 15.6 Hz, 1H), 7.40 (d, *J* = 15.5 Hz, 1H), 7.22 (d, *J* = 7.2 Hz, 1H), 7.18–7.08 (m, 3H), 6.96 (d, *J* = 8.2 Hz, 1H), 6.84 (s, 2H), 6.18 (s, OH), 3.94 (OCH_3_), 3.85 (OCH_3_), 3.82 (OCH_3_). ^13^C-NMR (75 MHz, Chloroform-d) δ 189.78 (C = O), 153.74 (Ar-O), 151.73 (Ar-O), 148.38 (Ar-O), 146.91 (Ar-O), 145.05 (C = C), 142.18 (Ar), 137.10 (Ar), 129.03 (2C Ar), 128.19 (C = C), 127.57 (Ar), 126.67 (2C Ar), 126.58 (C = C), 125.99 (C = C), 123.41 (Ar), 119.62 (Ar), 114.98 (Ar), 114.54 (Ar), 112.31 (Ar), 111.81 (Ar), 110.16 (Ar), 56.25 (OCH_3_), 56.04 (OCH_3_), 55.85 (OCH_3_). HRMS-ESI (*m*/*z*): 417,1696 [M+H]^+^ calcd. for C_26_H_25_O_5_ [M + H]^+^ 417.1623

(E)-1-(4-((E)-2,6-Dimethoxystyryl)phenyl)-3-(4-hydroxy-3-methoxyphenyl)prop-2-en-1-one (**3g**)

Yield 35%; m.p. 87–90 °C; IR (cm^−1^): ν_max_ 3365 (OH), 1649 (C = O), 1573 (C = C), 1510 (C = C_Ar_), 1249 (C-O-C); ^1^H-NMR (300 MHz, CDCl_3_) δ 8.01 (d, *J* = 8.0 Hz, 2H), 7.76 (d, *J* = 15.6 Hz, 1H), 7.60–7.66 (m, 4H), 7.41 (d, *J* = 15.6 Hz, 1H), 7.25–7.17 (m, 2H), 7.14 (s, 1H), 6.96 (d, *J* = 8.2 Hz, 1H), 6.60 (d, *J* = 8.3 Hz, 2H), 6.12 (s, OH), 3.95 (s, 3H), 3.91 (s, 6H). ^13^C-NMR (75 MHz, Chloroform-d) δ 189.86 (C = O), 158.91 (2 Ar-O), 148.20 (Ar-O), 146.82 (Ar-O), 144.73 (C = C), 143.92 (Ar), 136.64 (Ar), 131.13 (C = C), 128.94 (2 Ar), 128.06 (Ar), 127.69 (Ar), 126.44 (2 Ar), 123.33 (C = C), 122.74 (Ar), 119.87 (C = C), 114.89 (Ar), 114.28 (Ar), 110.06 (Ar), 103.97 (2 Ar), 56.06 (OCH_3_), 55.88 (2 OCH_3_). HRMS-ESI (*m*/*z*): 417,1696 [M+H]^+^ calcd. for C_26_H_25_O_5_ [M+H]^+^ 417.1623

(E)-1-(4-((E)-3,5-Dimethoxystyryl)phenyl)-3-(4-hydroxy-3-methoxyphenyl)prop-2-en-1-one (**3h**)

Yield 35%; m.p. 49–51 °C; IR (cm^−1^): ν_max_ 3388 (OH), 1651 (C = O), 1577 (C = C), 1508 (C = C_Ar_), 1267 (C-O-C); ^1^H-NMR (300 MHz, CDCl_3_) δ 8.00 (d, *J* = 8.4 Hz, 2H), 7.76 (d, *J* = 15.5 Hz, 1H), 7.58 (d, *J* = 8.4 Hz, 2H), 7.39 (d, *J* = 15.5 Hz, 1H), 7.21 (dd, *J* = 8.2, 2.0 Hz, 1H), 7.16–7.08 (m, 3H), 6.96 (d, *J* = 8.2 Hz, 1H), 6.69 (d, *J* = 2.2 Hz, 3H), 6.42 (t_app_, *J* = 2.2 Hz, 2H), 3.93 (OCH_3_), 3.82 (2 OCH_3_). ^13^C-NMR (75 MHz, Chloroform-*d*) δ 189.69 (C = O), 161.04 (2 Ar-O), 148.49 (Ar-O), 146.97 (Ar-O), 145.18 (C = C), 141.43 (Ar), 138.77 (Ar), 137.34 (Ar), 131.26, 129.07 (2C Ar), 128.09 (C = C), 127.49 (C = C), 126.66 (2C Ar), 123.43 (C = C), 119.46 (Ar), 115.04 (Ar), 110.24 (Ar), 104.91 (2C Ar), 100.59 (Ar), 56.04 (OCH_3_), 55.44 (2 OCH_3_). HRMS-ESI (*m*/*z*): 417,1696 [M+H]^+^ calcd. for C_26_H_25_O_5_ [M + H]^+^ 417.1623

(E)-1-(4-((E)-3,4-dimethoxystyryl)phenyl)-3-(4-hydroxy-3-methoxyphenyl)prop-2-en-1-one (**3i**)

Yield 55%; m.p. 67–70 °C; IR (cm^−1^): ν_max_ 3390 (OH), 1649 (C = O), 1577 (C = C), 1508 (C = C_Ar_), 1265 (C-O-C); ^1^H-NMR (600 MHz, CDCl_3_) δ 8.01 (d, *J* = 8.3 Hz, 2H), 7.77 (d, *J* = 15.5 Hz, 1H), 7.60 (d, *J* = 8.3 Hz, 2H), 7.40 (d, *J* = 15.5 Hz, 1H), 7.23 (dd, *J* = 8.2, 1.5 Hz, 1H), 7.19 (d, *J* = 16.2 Hz, 1H), 7.13 (d, *J* = 1.5 Hz, 1H), 7.11–7.07 (m, 2H), 7.02 (d, *J* = 16.2 Hz, 1H), 6.96 (d, *J* = 8.2 Hz, 1H), 6.88 (d, *J* = 8.2 Hz, 1H), 3.96 (OCH_3_), 3.96 (OCH_3_), 3.91 (OCH_3_). ^13^C-NMR (75 MHz, Chloroform-d) δ 189.68 (C = O), 149.51 (Ar-O), 149.22 (Ar-O), 148.32 (Ar-O), 146.86 (Ar-O), 145.00 (C = C), 141.95 (Ar), 136.98 (Ar), 131.17 (Ar), 129.89 (C = C), 129.10 (2C Ar), 127.60 (C = C), 126.33 (2C Ar), 125.67 (Ar), 123.37 (C = C), 120.56 (Ar), 119.64, 114.94 (Ar), 111.22 (Ar), 110.16 (Ar), 108.85 (Ar), 56.07 (OCH_3_), 56.01 (OCH_3_), 55.95 (OCH_3_). HRMS-ESI (*m*/*z*): 417,1696 [M+H]^+^ calcd. for C_26_H_25_O_5_ [M + H]^+^ 417.1623

(E)-3-(4-Hydroxy-3-methoxyphenyl)-1-(4-((E)-4-hydroxy-3-methoxystyryl)phenyl)prop-2-en-1-one (**3j**)

Yield 21%; m.p. 142–146 °C; IR (cm^−1^): ν_max_ 3232 (OH), 1641 (C = O), 1589 (C = C), 1508 (C = C_Ar_), 1269 (C-O-C); ^1^H-NMR (300 MHz, CDCl_3_) δ 8.01 (d, *J* = 8.3 Hz, 2H), 7.77 (d, *J* = 15.5 Hz, 1H), 7.58 (d, *J* = 8.3 Hz, 2H), 7.40 (d, *J* = 15.5 Hz, 1H), 7.22 (dd, *J* = 8.3, 1.9 Hz, 1H), 7.15–7.10 (m, 1H), 7.08–7.03 (m, 2H), 6.99 (d, *J* = 9.8 Hz, 1H), 6.93 (d, *J* = 8.3 Hz, 1H), 6.10 (s, OH), 5.88 (s, OH), 3.95 (s, 2 OCH_3_). ^13^C-NMR (75 MHz, Chloroform-d) δ 189.70 (C = O), 148.34 (Ar-O), 146.88 (Ar-O), 146.86 (Ar-O), 146.25 (Ar-O), 145.03 (C = C), 142.01 (Ar), 136.87 (Ar), 131.32 (Ar), 129.45 (C = C), 129.10 (2C Ar), 127.58 (C = C), 126.27 (2C Ar), 125.32 (C = C), 123.36 (Ar), 121.08 (Ar), 119.57 (Ar), 114.96 (Ar), 114.75 (Ar), 110.19 (Ar), 108.48 (Ar), 56.06 (OCH_3_), 55.98 (OCH_3_). HRMS-ESI (*m*/*z*): 403,1540 [M+H]^+^ calcd. for C_25_H_23_O_5_ [M + H]^+^ 403.1467

(E)-3-(4-Hydroxy-3-methoxyphenyl)-1-(4-((E)-3,4,5-trimethoxystyryl)phenyl)prop-2-en-1-one (**3k**)

Yield 55%; m.p. 74–77 °C; IR (cm^−1^): ν_max_ 3390 (OH), 1651 (C = O), 1579 (C = C), 1504 (C = C_Ar_), 1265 (C-O-C); ^1^H-NMR (600 MHz, CDCl_3_) δ 8.03 (d, *J* = 8.2 Hz, 2H), 7.77 (d, *J* = 15.6 Hz, 1H), 7.62 (d, *J* = 8.1 Hz, 2H), 7.41 (d, *J* = 15.6 Hz, 1H), 7.24 (dd, *J* = 8.2, 1.9 Hz, 1H), 7.17 (d, *J* = 16.2 Hz, 1H), 7.14 (d, *J* = 1.9 Hz, 1H), 7.06 (d, *J* = 16.2 Hz, 1H), 6.97 (d, *J* = 8.2 Hz, 1H), 6.78 (s, 2H), 3.97 (OCH_3_), 3.93 (2 OCH_3_), 3.89 (OCH_3_). ^13^C-NMR (75 MHz, Chloroform-d) δ 189.69 (C = O), 153.51 (2 Ar-O), 148.35 (Ar-O), 146.87 (Ar-O), 145.12 (C = C), 141.56 (Ar), 137.28 (Ar-O), 132.54 (Ar), 131.28 (Ar), 129.11 (2C Ar), 127.57 (C = C), 127.09 (C = C), 126.51 (2C Ar), 123.37 (C = C), 119.61 (Ar), 114.95 (Ar), 110.18 (Ar), 103.90 (2C Ar), 61.06 (OCH_3_), 56.20 (OCH_3_), 56.08 (OCH_3_). HRMS-ESI (*m*/*z*): 447,1803 [M+H]+ calcd. for C_27_H_27_O_6_: 447.1729

(E)-1-(4-((E)-4-Hydroxy-3,5-dimethoxystyryl)phenyl)-3-(4-hydroxy-3-methoxyphenyl)prop-2-en-1-one (**3l**)

Yield 40%; m.p. 86–88 °C; IR (cm^−1^): ν_max_ 3332 (OH), 1641 (C = O), 1589 (C = C), 1508 (C = C_Ar_), 1269 (C-O-C); ^1^H-NMR (300 MHz, CDCl_3_) δ 8.02 (d, *J* = 8.3 Hz, 2H), 7.77 (d, *J* = 15.6 Hz, 1H), 7.59 (d, *J* = 8.3 Hz, 2H), 7.40 (d, *J* = 15.6 Hz, 1H), 7.19–7.12 (dd, *J* = 8.3, 1.8 Hz, 1H), 7.16 (d, *J* = 16.0 Hz, 1H), 7.14–7.11 (m, 1H), 7.00 (d, *J* = 16.0 Hz, 2H), 6.95 (d, *J* = 8.3 Hz, 1H), 6.79 (s, 2H), 6.02 (s, OH), 5.69 (s, OH), 3.96 (OCH_3_), 3.95 (2 OCH_3_). ^13^C-NMR (75 MHz, Chloroform-d) δ 189.64 (C = O), 148.31 (Ar-O), 147.29 (2 Ar-O), 146.85 (Ar-O), 145.02 (C = C), 141.82 (Ar-O), 136.99 (Ar), 135.41 (Ar), 131.49 (Ar), 129.10 (2C Ar), 128.42 (C = C), 127.59 (C = C), 126.30 (2C Ar), 125.69 (Ar), 123.34 (C = C), 119.59 (Ar), 114.93 (Ar), 110.17 (Ar), 103.69 (2C Ar), 56.38 (2 OCH_3_), 56.07 (OCH_3_). HRMS-ESI (*m*/*z*): 433,1646 [M+H]^+^ calcd. for C_26_H_25_O_6_: 433.1572.

### 4.2. Biological Activity Assays

#### 4.2.1. Cell Lines and Culture Medium

Biological assays were performed using an adenocarcinoma colon cancer cell line (SW480), its metastatic derivative (SW620) and non-malignant cells (CHO-K1). These were obtained from The European Collection of Authenticated Cell Cultures (ECACC, England) and maintained in Dulbecco’s Modified Eagle Medium, supplemented with 10% heat-inactivated (56 °C) horse serum, 1% penicillin/streptomycin and 1% non-essential amino acids (Gibco Invitrogen, Carlsbad, USA). For all experiments, horse serum was reduced to 3%, and the medium was supplemented with 5 mg/mL transferrin, 5 ng/mL selenium and 10 mg/mL insulin (ITS-defined medium; Gibco, Invitrogen, Carlsbad, CA, USA) [28].

#### 4.2.2. Cytotoxic Activity

Cytotoxicity of the synthesized hybrids, lead and reference compounds was evaluated through Sulforhodamine B (SRB) assay, a colorimetric test that is based on staining of total cellular protein of adherent cells. Cells were seeded to a final density of 20,000 cells/well in 96-well tissue culture plates and incubated at 37 °C in a humidified atmosphere at 5% CO_2_. All cultures were allowed to grow for 24 h, and afterward they were treated with DMSO (vehicle control 1%) or increasing concentrations (0.01–200 µM) of the synthesized hybrids, as well as curcumin and resveratrol (lead compounds), the equimolar mixture of these and 5-fluorouracil (5-FU; the standard drug). After treatment, cells were fixed with trichloroacetic acid (50% *v*/*v*) (MERCK) for a period of 1 h at 4 °C. Cell proteins were determined by staining with 0.4% (*w*/*v*) SRB (Sigma-Aldrich, United States), and then they were washed with 1% acetic acid for the removal of unbound SRB and left for air-drying. Protein bound SRB was solubilized in 10 mM Tris-base and the absorbance was measured at 492 nm in a microplate reader (Mindray MR-96A) [46]. All experiments were performed at least in triplicate.

#### 4.2.3. Antiproliferative Activity

Antiproliferative effect of the most active compounds was also tested through Sulforhodamine B (SRB) assay. Briefly, cells were seeded to a final density of 2500 cells/well in 96-well tissue culture plates and incubated in the same conditions described for cytotoxicity. Cultures were allowed to grow for 24 h and then were treated with increasing concentrations of the selected hybrids (10–160 µM, ranges dependent on the IC_50_ values) or DMSO (vehicle control, 1%), for 0, 2, 4, 6 and 8 days. Culture media was replaced every 48 h. After each incubation time, cells were fixed, stained and read as previously described for this technique [37].

#### 4.2.4. Statistical Analysis

All experiments were performed at least three times. Data are reported as mean ± SE (standard error). Statistical differences between control group (non-treated) and treated cells were evaluated by one-way ANOVA followed by the Dunnett’s test. Values with *p* ≤ 0.05 were considered significant. Data were analyzed with GraphPad Prism version 7.04 for Windows (Graph Pad Software, San Diego, CA, USA).

## Figures and Tables

**Table 1:** Cytotoxic effect of the hybrids against SW480, SW620 and CHO-K1 cell lines at 24 and 48 h.

Hybrid Compound	Structure	24 h					48 h				
		CHO-K1 IC_50_ (µM)	SW480 IC_50_ (µM)	SI	SW620 IC_50_ (µM)	SI	CHO-K1 IC_50_ (µM)	SW480 IC_50_ (µM)	SI	SW620 IC_50_ (µM)	SI
**3a**	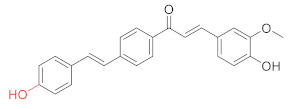	122.43 ± 15.5	95.96 ± 2.76	1.28	165.7 ± 11.78	0.74	51.58 ± 10.46	95.08 ± 7.02	0.54	20.15±1.21	2.56
**3b**	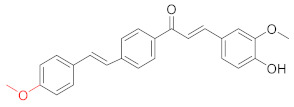	N.E.	N.E.	N.A.	N.E.	N.A.	N.E.	N.E.	N.A.	N.E.	N.A.
**3c**	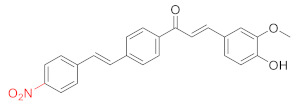	>3000	27.49 ± 2.00	>100	>3000	1.00	>3000	>200	>15	>3000	1.00
**3d**	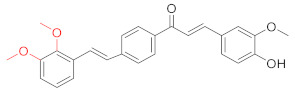	31 ± 2.93	34.15 ± 3.92	0.91	70.45 ± 3.8	0.44	12.4 ± 3.41	25.22 ± 3.54	0.49	26.05 ± 4.24	0.48
**3e**	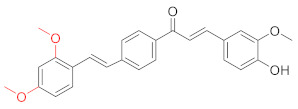	100.6 ± 1.00	54.37 ± 6.93	1.85	55.46 ± 8.0	1.81	43.27 ± 6.84	29.18 ± 4.36	1.48	20.44 ± 3.51	2.12
**3f**	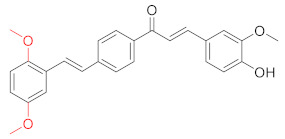	112.07 ± 15.8	84.37 ± 5.45	1.33	47.44 ± 10.53	2.36	23.89 ± 4.01	88.64 ± 1.84	0.27	27.62 ± 4.99	0.86
**3g**	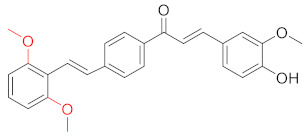	94.95 ± 1.67	8.55 ± 2.47	11.1	33.72 ± 4.20	2.82	32.38 ± 5.16	39.09 ± 5.68	0.83	27.6 ± 5.21	1.17
**3h**	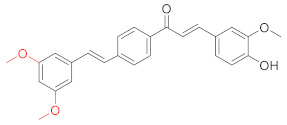	97.41 ± 2.62	47.21 ± 8.39	2.06	61.03 ± 12.76	1.60	23.17 ± 4.07	24.92 ± 1.65	0.93	21.36 ± 3.29	1.08
**3i**	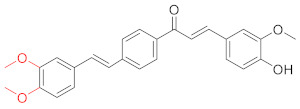	103.96 ± 5.03	49.7 ± 4.56	2.09	78.89 ± 6.20	1.32	28.29 ± 9.30	11.52 ± 2.78	2.46	29.33 ± 4.73	0.96
**3j**	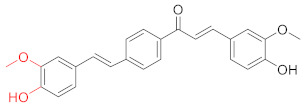	42.14 ± 4.85	7.98 ± 1.53	5.28	28.11 ± 4.27	1.50	24.28 ± 5.21	41.14 ± 7.80	0.59	28.8 ± 4.57	0.84
**3k**	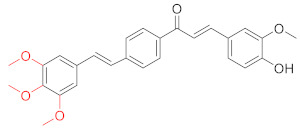	74.56 ± 4.20	88.5 ± 4.58	0.84	20.42 ± 2.43	3.65	29.35 ± 2.29	85.8 ± 1.27	0.34	11.81 ± 1.20	2.49
**3l**	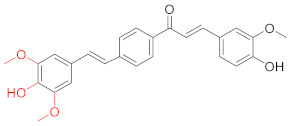	89.96 ± 8.55	63.47 ± 8.16	1.42	34.19 ± 5.09	2.63	24.84 ± 5.53	51.83 ± 5.85	0.48	29.75 ± 3.68	0.83
**Curcumin**		20.18 ± 2.41	84.64 ± 6.60	0.24	163.6 ± 5.88	0.12	36.29 ± 4.17	90.44 ± 1.00	0.40	22.21 ± 2.37	1.63
**Resveratrol**		118.4 ± 8.54	153.6 ± 10.64	0.77	549 ± 38.5	0.22	63.98 ± 8.92	123 ± 4.48	0.52	143.1 ± 4.03	0.45
**Mixture (Curcumin+Resveratrol)**		42.68 ± 4.03	35.05 ± 2.71	1.22	111.2 ± 3.38	0.38	19.93 ± 3.62	28.58 ± 2.67	0.69	25.81 ± 2.76	0.77
**5-FU**		543.5 ± 52.94	1544 ± 127.9	0.35	898.8 ± 60.74	0.59	173.2 ± 14.61	174.3 ± 19.1	0.99	180.9 ± 18.8	0.96

IC_50_ values were obtained from dose response curves for each compound. Selectivity index (SI) was calculated by the ratio of IC_50_ values in non-malignant CHO-K1 cells to IC_50_ of SW480 or SW620 cells. Data are presented as the mean ± SE of at least three independent experiments. N.E., Not Evaluated; N.A., Not Applicable.

**Table 2:** Data of viability (%) at the lowest and the highest concentrations evaluated.

Cell Line	Compound	Concentration (µM)	Time After Plating (Days)			
			2	4	6	8
			Viability (%) ± SE			
SW480	Hybrid 3e	10	53.9 ± 5.3	2.3 ± 0.3	3.8 ± 2.7	5.2 ± 0.3
		160	32.9 ± 1.3	0.9 ± 0.2	0.5 ± 0.2	0.1 ± 0.1
	Hybrid 3i	10	54.4 ± 7.4	2.0 ± 0.2	1.6 ± 0.2	1.6 ± 0.3
		160	30.3 ± 8.5	1.7 ± 0.1	0.1 ± 0.1	0.2 ± 0.1
SW620	Hybrid 3a	10	71.9 ± 1.9	10.7 ± 4.7	2.5 ± 0.5	0.2 ± 0.3
		160	16.1 ± 0.0	0	0	0
	Hybrid 3e	10	80.6 ± 4	11.1 ± 4.2	4.1 ± 2.2	0.6 ± 0.5
		160	5.0 ± 1	4.1 ± 0.0	0	0
	Hybrid 3k	10	56.1 ± 4.0	12.1 ± 0.0	1.9 ± 0.8	0.1 ± 0.1
		160	2.4 ± 0.7	0	0	0

## Sample Availability Statement

Samples of the hybrid compounds based on curcumin and resveratrol are available from the authors.
